# Brain-targeted delivery of PEGylated nano-bacitracin A against Penicillin-sensitive and -resistant Pneumococcal meningitis: formulated with RVG_29_ and Pluronic^®^ P85 unimers

**DOI:** 10.1080/10717544.2018.1486473

**Published:** 2018-11-07

**Authors:** Wei Hong, Zehui Zhang, Lipeng Liu, Yining Zhao, Dexian Zhang, Mingchun Liu

**Affiliations:** Key Laboratory of Zoonosis of Liaoning Province, College of Animal Science and Veterinary Medicine, Shenyang Agricultural University, Shenyang, P.R. China

**Keywords:** Pneumococcal meningitis, blood-brain barrier, rabies virus glycoprotein, Pluronic^®^ P85 unimers, RVG_29_-Nano-BA_P85_

## Abstract

Pneumococcal meningitis (PM), caused by *Streptococcus pneumonia*, remains a high-burden disease in developing countries. Antibiotic therapy has been limited due to the inefficiency of drug transport across the blood-brain barrier (BBB) and the emergence of drug-resistant strains. In our preliminary study, PEGylated nano-self-assemblies of bacitracin A (PEGylated Nano-BA_12K_) demonstrated a strong antibacterial potency against *S. pneumonia*. In this study, the potential application of this micelle for the treatment of both Penicillin-sensitive and -resistant PM was studied. To address BBB-targeting and -crossing issues, PEGylated Nano-BA_12K_ was formulated with a specific brain-targeting peptide (rabies virus glycopeptide-29, RVG_29_) and a P-glycoprotein inhibitor (Pluronic^®^ P85 unimers) to construct a mixed micellar system (RVG_29_-Nano-BA_P85_). RVG_29_-Nano-BA_P85_ demonstrated a strong antibacterial potency against 13 clinical isolates of *S. pneumonia*, even higher than that of Penicillin G, a conventional anti-PM agent. RVG_29_-Nano-BA_P85_ had more cellular uptake in brain capillary endothelial cells (BCECs) and higher BBB-crossing efficiency than single formulated Nano-BAs as shown in an *in vitro* BBB model. The enhanced BBB-permeability was attributed to the synergetic effect of RVG_29_ and P85 unimers through receptor-mediated transcytosis, exhaustion of ATP, and reduction in membrane microviscosity. *In vivo* results further demonstrated that RVG_29_-Nano-BA_P85_ was able to accumulate in brain parenchyma as confirmed by *in vivo* optical imaging. In addition, RVG_29_-Nano-BA_P85_ exhibited high therapeutic efficiencies in both Penicillin-sensitive and -resistant PM mouse models with negligible systemic toxicity. Collectively, RVG_29_-Nano-BA_P85_ could effectively overcome BBB barriers and suppressed the growth of both drug-sensitive and -resistant *S. pneumonia* in the brain tissues, which demonstrated its potential for the treatment of PM.

## 1. Introduction

Pneumococcal meningitis (PM) is a serious and life threatening disease in children and adolescents, which causes debilitating neurological symptoms, acute fatalities, and even long-term neurological sequelae in some survivors (Van De Beek et al., 2006; Weisfelt et al., 2006; Wilder-Smith, 2007; Brouwer et al., 2010). Despite the development of many new antibacterial agents, the fatality rate of PM remains high due to poor penetration across the blood-brain barrier (BBB) (Couchman, 1996; Egleton & Davis, 1997; Alam et al., 2010). In addition, the increasing number of resistant *S. pneumonia* isolates represents another therapeutic challenge. Therefore, it is of significance to develop an antibacterial agent against *S. pneumonia*, which can easily cross the BBB and less likely induce bacterial resistance.

Recently, many efforts have been devoted to overcome BBB. As is reported previously, the exploitation of nanocarriers for drug delivery has emerged as a promising alternative to conquer these problems. However, the effective transport of nanocarriers to the brain is still limited. Receptor-medicated transcytosis (RMT) is one of the most common strategies for nanocarriers to penetrate BBB (Zhou et al., 2010). Nanocarriers decorated with specific ligands can efficiently bind to the corresponding receptors overexpressed on BBB and then initiate the RMT process, resulting in the enhancement of drug penetration across the barriers and accumulation in the brain. Various ligands or monoclonal antibodies, such as insulin, transferrin, and OX26, have been intensively studied for the brain-targeted strategy, which exhibit obvious brain targeting properties when conjugated to nanocarriers (Smith & Gumbleton, 2006; Chen et al., 2015; Bi et al., 2016; Cui et al., 2016; Yin et al., 2016). Recently, a 29-amino-acid peptide derived from the rabies virus glycoprotein (RVG_29_) has been employed as a brain-targeted ligand for the RMT-based system, which can specifically bind to acetylcholine receptor (nAchR) on neuronal cells (Rapoport, 2001; Kim et al., 2006; Hwang et al., 2011). RVG_29_ is a promising brain-targeting ligand due to its relatively small molecular weight, high synthesizability, relatively low cytotoxicity, and immunogenicity (Lo & Wang, 2008). Lee et al. have demonstrated a higher accumulation of miR-124a in the brain parenchyma using RVG_29_ modified SSPEI polymeric nanocarrier compared to the accumulation of miR-124a encapsulated SSPEI without the RVG_29_ peptide (Hwang et al., 2011).

Another emerging strategy to enhance drug delivery to the central nervous system is the co-administration of a pharmacological modulator or a formulation component that blocks P-gp-mediated drug efflux. Both *in vitro* and *in vivo* studies have demonstrated that Pluronic block copolymers, such as P85, can inhibit the P-gp drug efflux system and increase the permeability of a broad spectrum of drugs across the BBB (Miller et al., 1997). Pluronics can influence mitochondria function and energy conservation, resulting in depletion of the intracellular ATP. Since the drug efflux transporters require an expenditure of cellular energy, the effects of Pluronics on intracellular ATP levels can reduce the drug efflux. In addition to energy depletion, Pluronics can also decrease membrane fluidization contributing to the inhibition of P-gp efflux function. Thus, Pluronic block copolymers have a ‘double-punch’ effect in BBB: (i) effect on the energy conservation and (ii) effect on membrane fluidization, both of which have a combined potent inhibition of P-gp function. Remarkably, these effects are most apparent at the copolymer concentrations below their critical micellization concentration (CMC), suggesting that the Pluronics unimers are responsible for the BBB permeabilization. This is attributed to the alternatives in the structure of the lipid bilayers as a result of immersion of hydrophobic PPO chains into the biomembrane hydrophobic areas (Regev et al., 1999).

**Figure 1.**
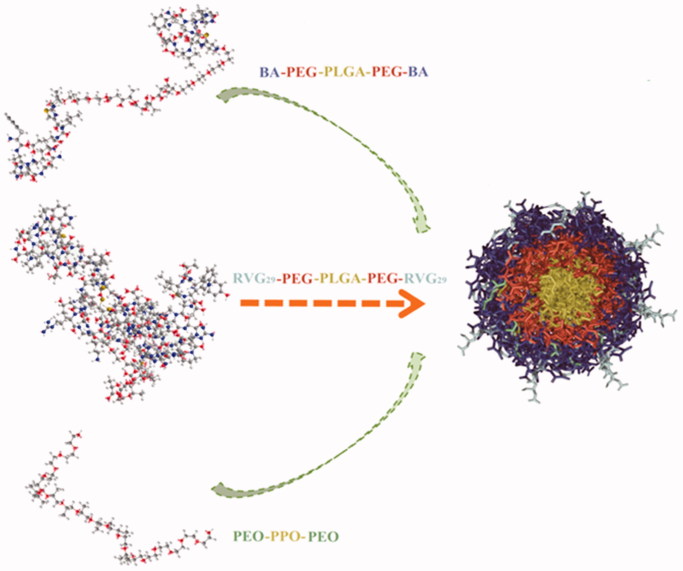
Schematic diagram of the brain-targeting mixed micellar delivery system (RVG_29_-Nano-BA_P85_).

In our previous study, the PEGylated Nano-BA_12K_ based mainly on BA-PEG-PLGA_12K_-PEG-BA demonstrated strong antibacterial activities against a broad spectrum of bacteria, including *S. pneumonia*, yet induced relatively lower toxicity both *in vitro* and *in vivo* (Hong et al., 2018). The objective of the present research was to evaluate the possibility of using PEGylated Nano-BA_12K_ to treat both Penicillin-sensitive and -resistant PM. In order to efficiently transport across BBB, a mixed micelles (RVG_29_-Nano-BA_P85_) based mainly on BA-PEG-PLGA_12K_-PEG-BA and RVG_29_-PEG-PLGA-PEG-RVG_29_, loading with Pluronic^®^ P85 unimers, were constructed (Figure 1). BA-PEG-PLGA_12K_-PEG-BA was employed for high antibacterial efficiency. The exposure of the RVG_29_ ligand at the periphery of the RVG_29_-Nano-BA_P85_ was employed as a targeting ligand to the brain, while Pluronic^®^ P85 unimers could weaken P-gp function of BBB to further improve membrane translocation. The RVG_29_-Nano-BA_P85_ was expected to fulfill three sequential tasks: (1) precisely targeting the brain tissue and reducing the nephrotoxicity of bacitracin A; (2) clarifying the synergistic effect of action against both Penicillin-sensitive and -resistant *S. pneumonia*, since its unassembled counterpart BA did not possess such antibacterial potency; (3) providing theoretical basis for potential treatment of PM. The antibacterial potency and mechanism of RVG_29_-Nano-BA_P85_ against *S. pneumonia* (Penicillin-sensitive and Penicillin-resistant strains) was first explored through transmission electron microscope (TEM), atomic force microscope (AFM), fluorescence, and ultra-violence fluorescence. Then, to verify the brain targeting efficiency, *in vitro* cellular uptake, penetration efficiency across a BBB model and *in vivo* optical imaging were performed. Eventually, the therapeutic effects of RVG_29_-Nano-BA_P85_ were evaluated on both Penicillin-sensitive and -resistant PM mouse models.

## 2. Experimental

### 2.1 Materials

#### 2.1.1 Reagents

HO-PEG(2K)-PLGA(12K)-PEG(2K)-OH was purchased from Xi’an ruixi Biological Technology CO., Ltd. (Xi’an, China). Pluronic^®^ P85, bacitracin A, and Penicillin G were purchased from Sigma-Aldrich (Shanghai, China). The peptide RVG29 with a cysteine on the C-terminal (YTIWMPENPRPGTPCDIFTNSRGKRASNGC) was synthesized by GL Biochem Ltd (Shanghai, China). ATP assay kit and enhanced BCA Protein Assay Kit were purchased from Beyotime Biotechnology Co., Ltd. (Nantong, China). All the other reagents and chemicals were of analytical or chromatographic grade and were purchased from Concord Technology (Tianjing, China).

#### 2.1.2 Bacteria

*Streptococcus pneumonia* ATCC49619 was purchased from American Type Culture Collection (Manassas, VA, USA). The 12 additional strains for susceptibility testing are clinical isolates of *S. pneumonia* from cerebrospinal fluid (CSF) of meningitis patients, which were obtained from the First Hospital of China Medical University (Shenyang, China) and stored at −80 °C in 40% (v/v) glycerol prior to use.

#### 2.1.3 Cell lines

Brain capillary endothelial cells (BCECs) were kindly provided by Prof. Qin J. (School of pharmacy, Fudan University), which were obtained from Cell Bank, Chinese Academy of Sciences (Shanghai, China) (Chen et al., 2017). Culture plates and dishes were purchased from Corning Inc. (New York, NY). BCECs were cultured in special Dulbecco’s modified Eagle medium (DMEM, Sigma-Aldrich) supplemented with 15% heat-inactivated fetal bovine serum (FBS), 100 U/mL penicillin, and 100 µg/mL streptomycin. All the cells were cultured at 37 °C in a humidifier with 5% CO_2_ atmosphere. All the experiments were performed on the cells in the logarithmic phase of growth.

#### 2.1.4 Animals

Male Kunming (KM) mice, weighing from 20 to 25 g, were used in the experiment. KM mice were supplied by Liaoning Changsheng Biotechnology Co., Ltd. (Benxi, China), and acclimated at 25 °C and 55% of humidity under natural light/dark conditions. All animal experiments were carried out in accordance with the guidelines evaluated and approved by the ethics committee of Shenyang Agricultural University (Shenyang, China).

#### 2.1.5 Tested formulations

RVG_29_-Nano-BA_P85_: RVG_29_-mediated mixed micelles composed of BA-PEG-PLGA_12K_-PEG-BA, RVG_29_-PEG-PLGA_12K_-PEG-RVG_29_, and Pluronic^®^ P85; Nano-BA_P85_: mixed micelles composed of BA-PEG-PLGA_12K_-PEG-BA, PEG-PLGA_12K_-PEG, and Pluronic^®^ P85; RVG_29_-Nano-BA: RVG_29_-mediated mixed micelles composed of BA-PEG-PLGA_12K_-PEG-BA and RVG_29_-PEG-PLGA_12K_-PEG-RVG_29_; Nano-BA: mixed micelles composed of BA-PEG-PLGA_12K_-PEG-BA and PEG-PLGA_12K_-PEG; PEGlated Nano-BA_12K_: single micelles only composed of BA-PEG-PLGA_12K_-PEG-BA.

### 2.2 Synthesis and characterization of copolymers

All of the copolymers used in this work were homemade. The details of synthesis and characterization of RVG_29_-PEG-PLGA-PEG-RVG_29_ were shown in the Supplementary Data.

### 2.3 Preparation and characterization of nano-BAs

The RVG_29_-Nano-BA_P85_ was prepared by the thin-film hydration method. Briefly, 200 mg of copolymer mixtures consisting of BA-PEG-PLGA_12K_-PEG-BA, RVG_29_-PEG-PLGA_12K_-PEG-RVG_29_, and Pluronic^®^ P85 were dissolved in acetonitrile (30 mL), followed by rotary evaporation at 40 °C for 2 h to obtain a thin film. After maintained in a vacuum for another 12 h, the resultant thin film was hydrated with 20 mL of PBS (pH 7.4) at 35 °C for 1 h. The micellar solution was then filtrated through a 0.22 μm film. An identical operation was conducted to prepare Nano-BA_P85_ except that the equivalent weight ratio of RVG_29_-PEG-PLGA_12K_-PEG-RVG_29_ was substituted by PEG-PLGA_12K_-PEG. The RVG_29_-Nano-BA was prepared as described above without loading of Pluronic^®^ P85. For the preparation of Nano-BA, the copolymer mixtures only consisted of BA-PEG-PLGA_12K_-PEG-BA and PEG-PLGA_12K_-PEG.

The particle size and ξ potential of the mixed micelles (RVG_29_-Nano-BA_P85_, Nano-BA_P85_, RVG_29_-Nano-BA, and Nano-BA) were determined with a NICOMP^TM^ 380 ZLS (Santa Barbara, USA). The morphologies of the mixed micelles were observed under the transmission electron microscopy (TEM, Hitachi HT-7700, Hitachi, Shiga, Japan) without staining. Samples were prepared by dipping a copper grid into the micellular solution, and an additional solution was blotted away with a strip of filter papers. The solution was evaporated at room temperature for 2 h before TEM observation.

### 2.4 *In vitro* antibacterial activity assays

The minimal inhibitory concentrations (MICs) of RVG_29_-Nano-BA_P85_, RVG_29_-Nano-BA_,_ Nano-BA_P85_, Nano-BA, PEGylated Nano-BA_12K_ and BA against 13 isolates of *S. pneumonia* strains were determined using a modified standard micro-dilution method as previously reported (Dong et al., 2012; Ma et al., 2012). Penicillin G was selected as the positive control, while MHB was used as the negative control.

### 2.5 Evaluation of BBB targeting ability of RVG_29_-nano-BA_P85_

#### 2.5.1 *In vitro* cellular uptake

The BCECs were seeded on a cover-slide system at a density of 2.5 × 10^4^ cells/well in a humidifier with 5% CO_2_ atmosphere overnight at 37 °C. Then, the cells were treated with FITC-labeled BA, FITC-loaded RVG_29_-Nano-BA_P85_, Nano-BA_P85_, RVG_29_-Nano-BA, Nano-BA, and PEGylated Nano-BA_12K_, respectively. After incubation for 2 h, the cells were washed three times with the cold PBS and stained with 10 μM Hoechst 33258 (10 min) to visualize nuclei. Then, the cells were fixed with 4% paraformaldehyde for 30 min. The microscope images were captured using a confocal laser scanning microscope (CLSM, Leica SP8, Leica, Wetzlar, Germany).

For quantitative analysis of the cellular uptake of the tested formulations (BA solution, RVG_29_-Nano-BA_P85_, Nano-BA_P85_, RVG_29_-Nano-BA, Nano-BA and PEGylated Nano-BA_12K_), BCECs were seeded at a density of 1.0 × 10^5^ cells/well in 6-well plates and incubated for 24 h to allow cell attachment. After 24 h, the medium was replaced with cell culture medium containing tested formulations, respectively. After 2 h of incubation, the cells were washed three times with PBS. The cells were then harvested by trypsinization, centrifuged at 1000 rpm for 5 min, re-suspended in 500 μL of PBS medium, and analyzed using a flow cytometer (BD FACS Aria^TM^, USA). BCECs cultured under normal conditions served as the control. Living cells were defined by gating the major population of the cells, and only cells within this gate were analyzed. The mean fluorescence intensity of the cells was calculated using the histogram plot.

#### 2.5.2 Transport studies across BCEC monolayers

The BCECs were seeded at a density of 2 × 10^4^ cells/well onto polycarbonate 24-well Transwell filters of 1.0 μm mean pore size, and 0.33 cm^2^ surface area. After culturing about 72 h, cells were checked for complete confluence under the microscope. The cell monolayer integrity was monitored using an epithelial voltammeter (EVOM^2^, World Precision Instruments, USA) to measure the transendothelial electrical resistance (TEER). Only cell monolayers with TEER above 150 Ω cm^2^ were selected for the following experiments.

A total of 250 μL of fetal bovine serum (FBS)-free cell culture medium containing 0.02% sodium fluorescein was introduced to the donor chamber at the starting time to monitor the integrity of BCEC monolayers. At the same time, BCEC monolayers were treated with the tested formulations (FITC-loaded RVG_29_-Nano-BA_P85_, Nano-BA_P85_, Nano-BA, RVG_29_-Nano-BA, and PEGylated Nano-BA_12K_, respectively). Cells were then incubated on a platform, shaking at 50 rpm at 37 °C. A total of 20 μL of sample was extracted from the receiving chamber on each time point and 20 μL of FBS-free cell culture medium was added to the receiving chamber for compensation. The amount of FITC-loaded formulations appearing in the receiving chamber was measured by a multifunctional microplate reader (Tecan, Tecan Group Ltd., Switzerland). The apparent permeability (*P*_app_) was calculated according to the following Equation (1):

(1) $$P\text{app}\;=(\frac{\text{dQ}}{\text{dt}})\times \left(\frac{1}{Co}\right)\times (\frac{1}{A})$$

where, d*Q*/d*t* is the permeability rate (nmol/s), *C*_0_ is the initial concentration (nmol/mL) in the donor chamber, and *A* is the surface area (cm^2^) of the membrane filter.

Both TEER and the permeability of sodium fluorescein of BCEC monolayers were measured from time 0 to 4 h to monitor the integrity of the monolayers.

#### 2.5.3 ATP content assay

For the intracellular ATP assay, the confluent BCECs were treated with RVG_29_-Nano-BA_P85_, Nano-BA_P85_, Nano-BA, RVG_29_-Nano-BA, PEGylated Nano-BA_12K_, and Pluronic^®^ P85 unimers for 2 h. Then, the cells were washed twice with ice-cold PBS and solubilized in cell lysates followed by immediate centrifugation (12,000*g*) at 4 °C for 10 min. The supernatant was collected for ATP quantification using the kit based on the luciferin/luciferase assay. Light emission was measured with an Ultra-Weak luminescence Analyzer (Spark, Tecan Group Ltd., Switzerland). Raw data were converted to ATP concentrations according to the standard calibration. ATP contents were normalized by the protein content in each sample determined by a BCA kit, and each data point presented the mean ± SD of a minimum of six replicates. The blank medium was used as control.

#### 2.5.4 DPH labeling of BCECs

1,6-diphenyl-1,3,5-hexatriene (DPH) was used as a probe to examine the fluidity properties of the hydrocarbon region of the cell membrane after treated with RVG_29_-Nano-BA_P85_ (De Laat et al., 1977). DPH is a hydrophobic fluorescence compound that spontaneously incorporates in the hydrocarbon regions of the lipid membranes. Transferring DPH from the aqueous environment into the cell membranes results in a drastic increase of the intensity of the fluorescence emission of this probe. Furthermore, once the probe is incorporated in the lipid membranes, its fluorescence polarization is strongly dependent on the microenvironment. This probe provides valuable information concerning membrane structure, specifically, and membrane microviscosity. Briefly, after 24 h incubation, BCECs were washed twice with PBS and incubated with 2 µM DPH labeling solution for 1 h at 37 °C. Following the initial labeling with DPH, cells were washed twice with PBS to remove extracellular DPH and re-suspended in an appropriate volume of PBS. To evaluated the kinetic effects of tested formulations (RVG_29_-Nano-BA_P85_, Nano-BA_P85_, Nano-BA, RVG_29_-Nano-BA, and PEGylated Nano-BA_12K_), 30 µL of the tested formulation was added to 3 mL of cell suspension. Changes in membrane microviscosity were recorded immediately after addition of the tested formulation and up to 90 min following the addition.

### 2.6 *In vivo* biodistribution study

Approximately, 6-week-old male KM mice were used for the experiments. One day prior to inoculation (day 0) and during the period of the experiments, all mice received a daily intramuscular injection of cortisone acetate at 5.0 mg/kg. On day 1, the KM mice were sedated and intracisternally injected with 100 µL of 10^9^ CFU/mL of *S. pneumonia* ATCC 49619 and *S. pneumonia* 16167 mixture to establish the PM mouse model.

The *in vivo* distribution study was assessed using the *in vivo* FX Pro imaging system (Kodak, USA). The near-infrared (NIR) fluorescence dye DiR was selected for fluorescence imaging. Briefly, two days after infection, 0.2 mL of DiR-loaded RVG_29_-Nano-BA_P85_, Nano-BA_P85_, Nano-BA, RVG_29_-Nano-BA, and PEGylated Nano-BA_12K_ was intravenously injected into the PM mice through the tail vein, respectively. The time-dependent biodistribution in mice was imaged by Kodak *In Vivo* FX PRO Imaging System (Carestream Health, Inc., Rochester, NY) at time points of 2, 4, 8, 12, and 24 h post-injection. After 24 h, the mice were sacrificed and the major organs and thighs were harvested. Each organ was rinsed with physiological saline for three times followed by the capture of fluorescence images.

The content of BA in the brain was further quantified by a HPLC/MS/MS method. Two days after infection, the PM mice were injected via tail vein with formulations of BA solution, RVG_29_-Nano-BA_P85_, Nano-BA_P85_, Nano-BA, RVG_29_-Nano-BA, and PEGylated Nano-BA_12K_, respectively, with equivalent BA dose (30 mg/kg). At predetermined time points (2, 4, 8, 12, and 24 h), the mice were sacrificed; the infected brains were harvested and weighted; the BA content was quantitated by HPLC/MS/MS analysis.

### 2.7 *In vivo* anti-infective activity

The PM mouse model infected with *S. pneumonia* ATCC49619 and *S. pneumonia* 16167 was established as described above, respectively. The treatment was started on day 3 after inoculation and lasted for 11 consecutive days. The mice in the control group continued to receive no treatment, whereas the mice in the remaining groups were intravenously injected with RVG_29_-Nano-BA_P85_, Nano-BA_P85_, RVG_29_-Nano-BA, Nano-BA, PEGylated Nano-BA_12K_, and Penicillin G at the dose of 30 mg/kg per day. On day 3, 5, 8, 11, and 14, intracisternal taps were performed and approximately 0.5 mL of CSF was aspirated from each animal. The number of colony-forming unit (CFU) of *S. pneumonia* in the cerebrospinal fluid (CSF) was measured by serial dilution of CSF. The CSF samples were placed on blood agar plates at 37 °C for 36 h. The number of CFUs was counted and the results were expressed as l g CFU/mL of CSF. In addition, at day 14, brain tissue samples were taken, weighed, and homogenized. Then, 200 µL of homogenate was placed on blood agar plates and incubated at 37 °C for 36 h. The number of CFUs was counted at the end of culture and expressed as CFU/g of the brain tissues. The survival rate of mice was also monitored for 14 days post-infection.

### 2.8 Nephrotoxicity evaluation

Thirty-six of PM mice infected with *S. pneumonia* ATCC49619 and *S. pneumonia* 16167 were divided into six groups and given injections of saline, BA solution and RVG_29_-Nano-BA_P85_, respectively, at a dose of 30 mg/kg once a day for 11 days. Two days after the last administration, the mice were sacrificed. The histological sections of the kidney were stained with hematoxylin and eosin to evaluate the nephrotoxicity of different formulations.

### 2.9 Statistical analysis

All experiments were performed at least three times. Quantitative data are presented as the mean ± standard deviation (S.D). Statistical comparisons were determined by the analysis of variance (ANOVA) among ≥3 groups or Student’s *t-*test between the two groups. *p*-Values <.05 and <.01 were considered statistically significant.

## 3. Results

### 3.1 Composition of the RVG_29_-nano-BA_P85_

#### 3.1.1 The optimum content of RVG_29_-conjugated PEG-PLGA-PEG

**Figure 2.**
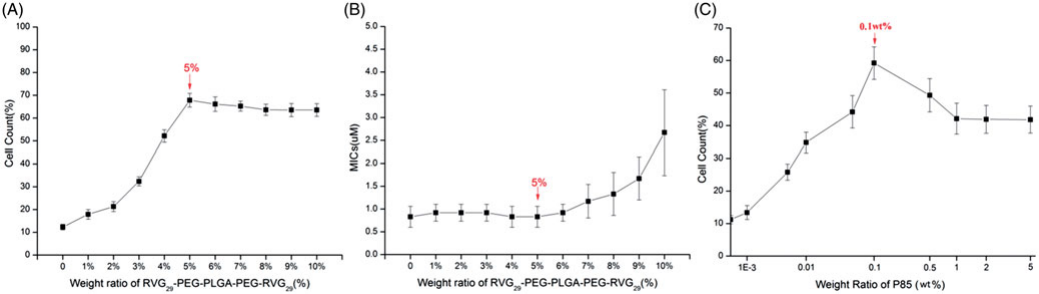
Cellular uptake (A) and antibacterial efficiency (B) of RVG_29_-Nano-BA_P85_ with different loading content of RVG_29_-PEG-PLGA-PEG-RVG_29_ (mean ± SD, *n* = 6). Flow cytometry analysis of the RVG_29_-Nano-BA_P85_ mixed micelles with different loading content of Pluronic**^®^** P85 taken up by BCECs (mean ± SD, *n* = 6) (C). The concentration of the copolymer mixture was 10 mg/mL.

RVG_29_-conjugated PEG-PLGA-PEG was employed to impart the possible active targeting of PEGylated Nano-BA_12K_ to brain parenchyma. RVG_29_ is an attractive targeting ligand which can efficiently pass through the BBB owning to the receptor-mediated endocytosis. The optimum loading content of RVG_29_-PEG-PLGA-PEG-RVG_29_ was evaluated on brain capillary endothelial cells (BCECs), which were widely used as a model for mimicking BBB (Bi et al., 2012; Xin et al., 2012). As shown in Figure 2, the cellular uptake of PEGylated Nano-BA_12K_ was enhanced by ligand modification in a ligand density-dependent manner. By increasing the amount of RVG_29_-PEG-PLGA-PEG-RVG_29_ in the lipid constitution, the cellular uptake was first improved, then slightly decreased and finally leveled off. The maximum cellular uptake occurred when the loading content of RVG_29_-PEG-PLGA-PEG-RVG_29_ was 5%. The *in vitro* antibacterial activity assay was also applied to investigate the optimum loading content of RVG_29_-PEG-PLGA-PEG-RVG_29_, since a large amount of RVG_29_-PEG-PLGA-PEG-RVG_29_ might reduce the antibacterial efficiency of RVG_29_-Nano-BA_P85_ against *S. pneumonia*. As illustrated in Figure 2(B), the MICs remained stable until the weight ratio of RVG_29_-PEG-PLGA-PEG-RVG_29_ in the lipid constitution beyond 6% (w/w), indicating that a small amount of RVG_29_-PEG-PLGA-PEG-RVG_29_ did not influence the antibacterial potency of RVG_29_-Nano-BA_P85_. Collectively, the mixed micelles composed of BA-PEG-PLGA-PEG-BA and RVG_29_-PEG-PLGA-PEG-RVG_29_ (95/5, w/w) displayed a desirable BBB transporting ability and antibacterial potency and were opted for the following studies.

#### 3.1.2 The effect of loading content of Pluronic^®^ P85 on BBB targeting ability

The effect of loading content of Pluronic**^®^** P85 unimers on the cellular uptake was evaluated on BCECs using flow cytometry (Figure 2(C)). The cellular uptake increased with the increase of Pluronic**^®^** P85 loading content until the loading content reached 0.1 wt%. Further increasing the loading content of Pluronic**^®^** P85 led to the decrease of the cellular uptake at first, and then, the cellular uptake leveled off.

Because the minor amount of Pluronic**^®^** P85 barely affected the antibacterial efficiency (data not shown), the mixed micelles RVG_29_-Nano-BA_P85_ consisting of 5 wt% RVG_29_-PEG-PLGA-PEG-RVG_29_ (0.5 mg/mL), 95 wt% BA-PEG-PLGA-PEG-BA (9.5 mg/mL), and 0.1 wt% Pluronic**^®^** P85 (0.01 mg/mL) were prepared for further studies.

### 3.3 Preparation and characterization of nano-BAs

**Figure 3.**
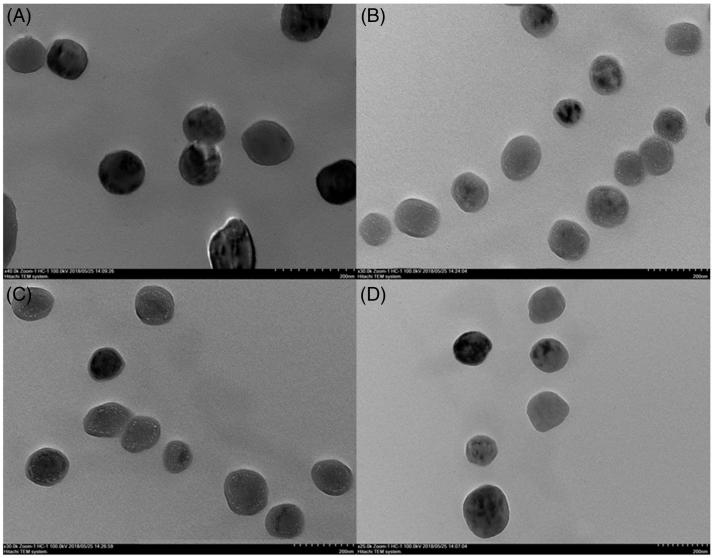
TEM images of RVG_29_-Nano-BA_P85_ (A), RVG_29_-Nano-BA (B), Nano-BA_P85_ (C), and Nano-BA (D).

We successfully prepared four types of Nano-BAs (RVG_29_-Nano-BA_P85_, RVG_29_-Nano-BA, Nano-BA_P85_, and Nano-BA). The mean particle sizes of various formulations ranged from 116 to 127 nm. PDIs were less than 0.1 and ξ potentials were around –4 mV (Figure S3 and Table S2). The incorporation of RVG_29_ and P85 did not affect the physical properties of Nano-BAs significantly. TEM micrograph (Figure 3) demonstrated that the mixed micelles exhibited a spherical shape and a smooth surface, forming homogenous nanostructures with the particle size in line with the DLS data. No significant changes of the particle size and PDI were observed in 10% FBS-contained medium over 24 h, suggesting good stability of the tested Nano-BAs in serum-contained medium (see Supporting Information).

### 3.4 *In vitro* antibacterial activities

Antibacterial activities of the Nano-BAs (RVG_29_-Nano-BA_P85_, RVG_29_-Nano-BA, Nano-BA_P85_, Nano-BA, and PEGylated Nano-BA_12K_) were determined against 13 isolates of *S. pneumonia* (Table S3). The growth of all the isolates was efficiently inhibited at a slightly lower concentration of RVG_29_-Nano-BA_P85_ than that of Penicillin G. Five Penicillin-resistant strains, *S. pneumonia* 16033, 16092, 16121, 16129, and 16167 (MIC ≥4 µM) were susceptible to RVG_29_-Nano-BA_P85_ with much lower MICs (1, 1, 0.5, 1, and 1 µM, respectively). For Penicillin-sensitive strains, there was no obvious difference in MICs among the tested Nano-BAs, indicating that a small amount of RVG_29_-PEG-PLGA-PEG-RVG_29_ and Pluronic^®^ P85 did not influence the antibacterial potency of Nano-BAs, which was in good correlation with the previous results. However, Penicillin-resistant strains were more susceptible to Nano-BAs loading with Pluronic^®^ P85 (RVG_29_-Nano-BA_P85_ and Nano-BA_P85_) than those without Pluronic^®^ P85 (RVG_29_-Nano-BA, Nano-BA, and PEGylated Nano-BA_12K_).

### 3.5 Effect of RVG_29_ and Pluronic^®^ P85 on targeting BCECs

#### 3.5.1 Cellular uptake on BCECs

**Figure 4.**
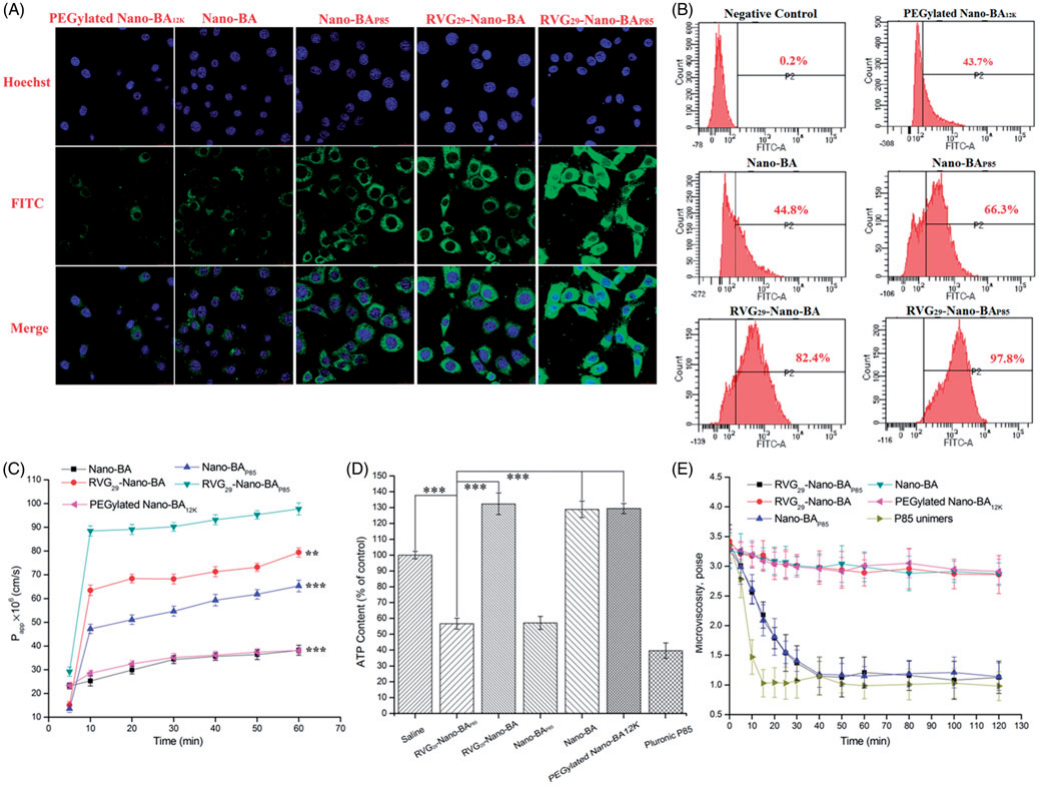
Qualitative (A) and quantitative (B) cellular uptake of FITC-labeled BA, FITC-loaded RVG_29_-Nano-BA_P85_, RVG_29_-Nano-BA, Nano-BA_P85_, Nano-BA, and PEGylated Nano-BA_12K_ on BCECs for 2 h. The permeability of FITC-labeled BA, FITC-loaded RVG_29_-Nano-BA_P85_, RVG_29_-Nano-BA, Nano-BA_P85_, Nano-BA, and PEGylated Nano-BA_12K_ across BCECs monolayer (C). The effects of RVG_29_-Nano-BA_P85_, RVG_29_-Nano-BA, Nano-BA_P85_, Nano-BA, PEGylated Nano-BA_12K_, and Pluronic^®^ P85 unimers on the intracellular ATP level (D) and microviscosity (E) of BCECs. *** *p* < .001, significance represents RVG_29_-Nano-BA_P85_ vs. RVG_29_-Nano-BA, Nano-BA_P85_, Nano-BA, and PEGylated Nano-BA_12K_. **p < .01; ***p < .001, significance represents RVG_29_-Nano-BA_P85_ vs. RVG_29_-Nano-BA, Nano-BA_P85_, Nano-BA, and PEGylated Nano-BA_12K_.

FITC-loaded Nano-BAs were used to investigate the cellular uptake characteristics, the results of which were shown qualitatively using fluorescent images. As shown in Figure 4(A), compared with the unmodified Nano-BAs, both RVG_29_-Nano-BA and Nano-BA_P85_ exhibited stronger green fluorescence signals inside BCECs, which indicated that formulation with the RVG_29_ modification and Pluronic^®^ P85 loading could effectively increase the uptake of Nano-BAs by BCECs. Furthermore, RVG_29_-Nano-BA_P85_ resulted in the strongest fluorescence signal among the tested groups, indicating that the synergetic effect of the receptor-mediated transcytosis and P-gp activity inhibition could significantly improve the cellular internalization of Nano-BAs. Similar results were obtained from flow cytometry (Figure 4(B)). Compared with Nano-BAs, the cellular uptake of RVG_29_-Nano-BA_P85_, RVG_29_-Nano-BA, and Nano-BA_P85_ was significantly increased on BCECs. Moreover, the fluorescence intensity of RVG_29_-Nano-BA_P85_ was 1.18- and 1.47-fold higher than those of RVG_29_-Nano-BA and Nano-BA_P85_, respectively, which was in good correlation with the CLSM result.

#### 3.5.2 Transport studies across BCEC monolayer

The results of transport studies were shown in Figure 4(C). *P*_app_ of RVG_29_-Nano-BA_P85_ was significantly higher than those of other tested formulations after 10 min, most significantly at 60 min. The transendothelial electrical resistance (TEER) showed no significant changes, assuring the integrity of BCEC monolayers during the experiments (data not shown).

#### 3.5.3 ATP depletion

To examine the effects of the loading P85 on cellular energy metabolism, the intracellular ATP content was measured using the luciferin-luciferase assay. The cells treated with blank medium were used as the control and the ATP level was normalized as 100%. As shown in Figure 4(D), when BCECs were treated with RVG_29_-Nano-BA_P85_ and Nano-BA_P85_, the ATP level dropped to 56.7% and 57.2% of the normal level, respectively. Treated with Pluronic^®^ P85 unimers, an addition of 18% decrease in the intracellular ATP level was observed. Compared to the P85 loading Nano-BAs, the ATP levels of BCECs treated with RVG_29_-Nano-BA, Nano-BA, and PEGylated Nano-BA_12K_ were increased to about 130%, which might be due to the energy-dependent endocytosis (Allen et al., 1999).

#### 3.5.4 Fluidity properties of the cell membranes

Interactions of Nano-BAs with BCEC membranes were evaluated in a fluorescence polarization study using DPH as a membrane probe. We examined the time-dependent changes in the fluorescence polarization of DPH of BCECs following exposure to the tested formulations (RVG_29_-Nano-BA_P85_, RVG_29_-Nano-BA, Nano-BA_P85_, Nano-BA, PEGylated Nano-BA_12K_, and Pluronic^®^ P85 unimers). As seen in Figure 4(E), there was a rapid decrease in the membrane microviscosity following addition of P85 unmiers to the cell suspension. After 20 min of exposure to P85 unimers, the microviscosity was leveled-off and then remained constant throughout the duration of the experiment. RVG_29_-Nano-BA_P85_ and Nano-BA_P85_ could also induce a similar decrease in the membrane microviscosity, but to a lesser degree compared with P85 unimers. RVG_29_-Nano-BA, Nano-BA, and PEGylated Nano-BA_12K_ exhibited limited effect on the membrane microviscosity of BCECs.

### 3.6 *In vivo* biodistribution study

**Figure 5.**
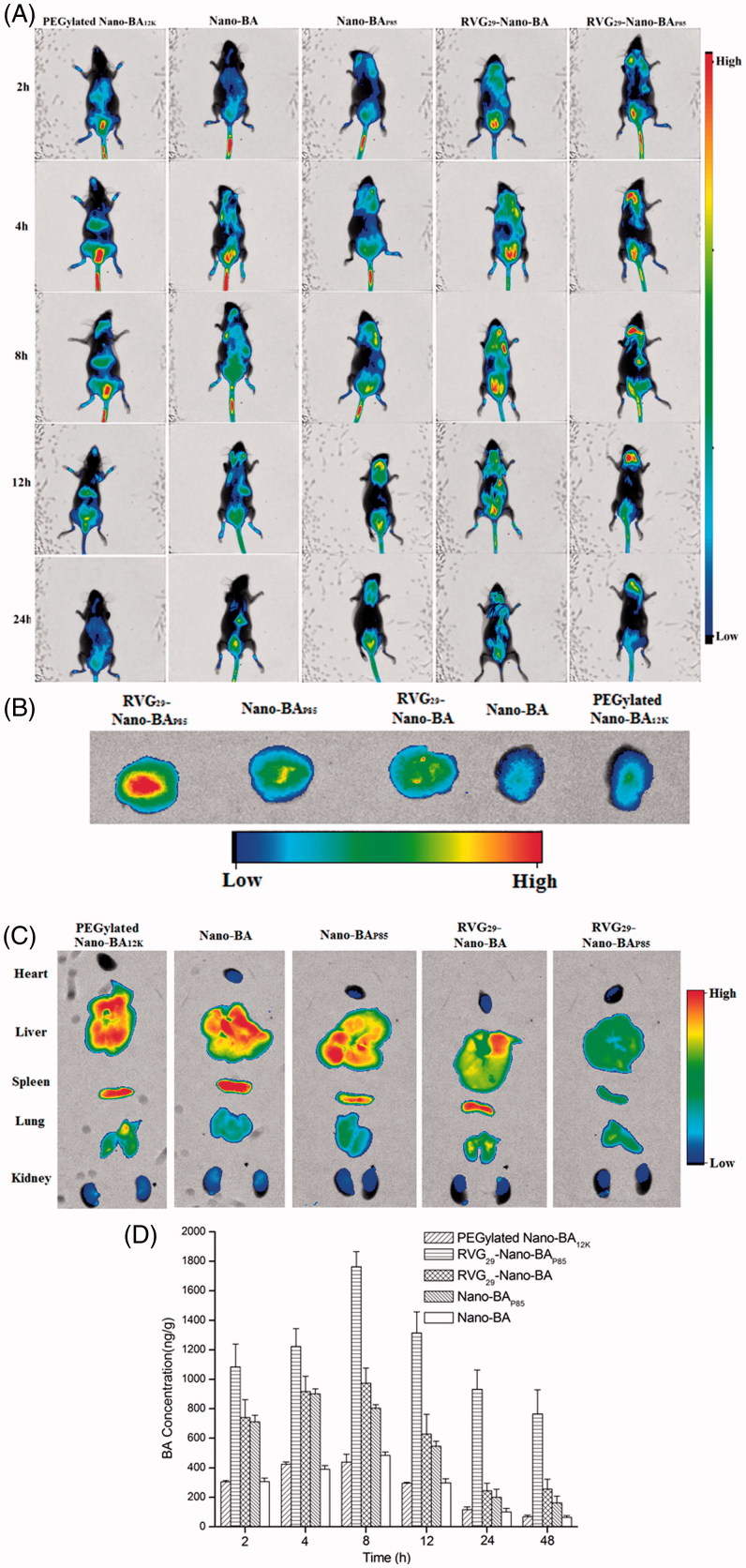
The *in vivo* noninvasive images of time-dependent whole body imaging of PM mice co-infected with *S. pneumonia* ATCC 49619 and *S. pneumonia* 16167 after *i.v.* injection of DiR-loaded RVG_29_-Nano-BA_P85_, RVG_29_-Nano-BA, Nano-BA_P85_, Nano-BA, and PEGylated Nano-BA_12K_ (A). The *ex vivo* optical images of the brain (B) and other organs (C) of the PM mice co-infected with *S. pneumonia* ATCC 49619 and *S. pneumonia* 16167 after *i.v.* injection of DiR-loaded RVG_29_-Nano-BA_P85_, RVG_29_-Nano-BA, Nano-BA_P85_, Nano-BA, and PEGylated Nano-BA_12K_. Quantitative analysis for accumulation in the brain of BA in the PM mice co-infected with *S. pneumonia* ATCC 49619 and *S. pneumonia* 16167 at different times after intravenous administration of RVG_29_-Nano-BA_P85_, RVG_29_-Nano-BA, Nano-BA_P85_, Nano-BA, and PEGylated Nano-BA_12K_ at a dose of 30 mg/kg, respectively (D).

In our study, we evaluated the *in vivo* brain targeting efficiency of different Nano-BAs containing near-infrared dye DiR in the coinfection PM mouse model. The Nano-BAs were administrated two days after infection, a real-time distribution of Nano-BAs was observed under Kodak *In Vivo* Imaging System FX PRO (Carestream Health, Inc,. Rochester, NY). As shown in Figure 5(A), the RVG_29_-Nano-BA_P85_ group exhibited the strongest fluorescence signal in the brain at all time points when compared with other tested formulations, indicating that the dual-formulation with RVG29 and P85 unimers could significantly facilitate RVG_29_-Nano-BA_P85_ to traverse the BBB and enhance the *in vivo* accumulation in brain tissues. The efficient BBB transporting capability and precise brain targeting ability of RVG_29_-Nano-BA_P85_ were further verified by *ex vivo* imaging of the brains after 24 h post-injection (Figure 5(B)). The RVG_29_-Nano-BA_P85_/DIR showed strongest fluorescence intensity in brain tissue among the tested formulations. Excised organs were also harvested and imaged (Figure 5(C)). The relatively intense fluorescence signal was observed in liver and spleen, corresponding to the main clearance route of nanoparticles (Tavano & Muzzalupo, 2016). No perspicuous difference in fluorescence signal of peripheric organs was observed among the groups, suggesting RVG_29_ modification and P85 loading would not change the nonspecific distribution behavior of Nano-BAs. In addition, RVG_29_-Nano-BA_P85_ resulted in the weak fluorescence signal in kidney, suggesting a low nephrotoxicity.

The amount of BA in brain tissues was quantified through a validated HPLC/MS/MS method (Figure 5(D)). RVG_29_-Nano-BA_P85_ showed effective time-dependent accumulation in brain tissues. The BA content increased from 2 h to 8 h, and reached the highest at 8 h. The relatively high content could last for more than 48 h. Compared to RVG_29_-Nano-BA_P85_, RVG_29_-Nano-BA, and Nano-BA_P85_ could also accumulate into the brain tissue, but to a lesser degree. Nano-BA and PEGylated Nano-BA_12K_ could not be internalized as much as functionalized Nano-BAs as determined by the minimal content in the brain.

### 3.7 *In vivo* antibacterial activity of RVG_29_-Nano-BA_P85_ in a *S. Pneumonia* meningitis mouse model

**Figure 6.**
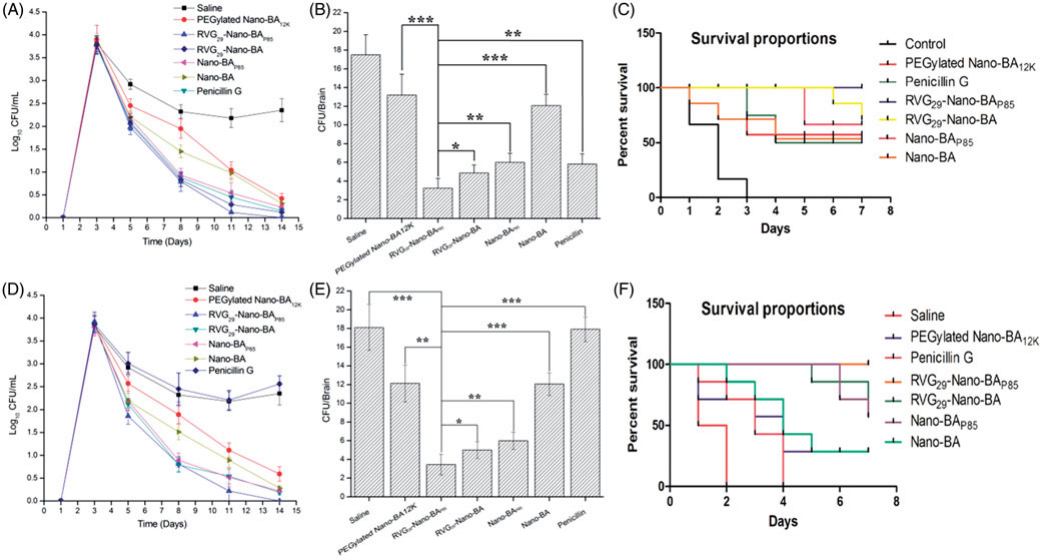
Colony formation unit of *S. pneumonia* ATCC 49619 in CSF of the mice without any treatment (the negative control group) or treated with Nano-BAs or Penicillin G for various periods of time (A). Quantitative counts of *S. pneumonia* ATCC 49619 per gram of brain tissues of the mice without any treatment (the control group) or treated with Nano-BAs or Penicillin G for 14 days (B). Survival rate of the PM mice infected with *S. pneumonia* ATCC49619 after treated with Saline, PEGylated Nano-BA12K, Penicillin G, RVG29-Nano-BAP85, RVG29-Nano-BA, Nano-BAP85, and Nano-BA, respectively (C). Colony formation unit of *S. pneumonia* 16167 in CSF of the mice without any treatment (the negative control group) or treated with Nano-BAs or Penicillin G for various periods of time (D). Quantitative counts of *S. pneumonia* 16167 per gram of brain tissues of the mice without any treatment (the control group) or treated with Nano-BAs or Penicillin G for 14 days (E). Survival rate of the PM mice infected with *S. pneumonia* 16167 after treated with Saline, PEGylated Nano-BA12K, Penicillin G, RVG29-Nano-BAP85, RVG29-Nano-BA, Nano-BAP85, and Nano-BA, respectively (F). Values are mean ± SD of six independent observations. *p < .05, **p < .01, ***p < .001, significance represents RVG29-Nano-BAP85 vs. RVG29-Nano-BA, Nano-BAP85, Nano-BA, and PEGylated Nano-BA12K.

With the promising *in vitro* results presented above, we proceeded to evaluate *in vivo* efficacy of RVG_29_-Nano-BA_P85_ in treating PM mice infected with *S. pneumonia* ATCC 49619 and *S. pneumonia* 16167, respectively. The antibacterial activities of the Nano-BAs in the subarachnoid space and brain parenchyma were studied over a defined period of time in comparison with Penicillin G. As shown in Figure 6(A), for *S. pneumonia* ATCC 49619 infected PM mice, all of the tested Nano-BAs and Penicillin G resulted in significant and continuous drop in bacterial counts in CSF during the course of the treatments that started on day 3, which was more obvious than those in the negative control group. Among the tested Nano-BAs, RVG_29_-Nano-BA_P85_ exhibited strongest ability in reduction of bacterial counts, even higher than that of Penicillin G group. In addition, brains were removed at 14 days post-infection to determine the CFUs in brain parenchyma. As shown in Figure 6(B), all of the Nano-BAs resulted in a significant decrease in bacterial counts. RVG_29_-Nano-BA_P85_ resulted in the eradication nearly 5.23 log_10_ viable CFU of *S. pneumonia*, which was much higher than that of Penicillin G (3.01) (*p* < .001). RVG_29_-Nano-BA reduced 3.59 log_10_ viable CFU of *S. pneumonia*, Nano-BA_P85_ reduced 2.91 log_10_ viable CFU of *S. pneumonia*, Nano-BA reduced 1.45 log_10_ viable CFU of *S. pneumonia*, while PEGylated Nano-BA_12K_ reduced 1.38 log_10_ viable CFU of *S. pneumonia*, indicating a partial protection against bacterial infection. BA solution has limited effect on the growth of *S. pneumonia*. The survival rates of mice infected with *S. pneumonia* treated with Penicillin G, RVG_29_-Nano-BA_P85_, RVG_29_-Nano-BA, Nano-BA_P85_, Nano-BA, and PEGylated Nano-BA_12K_ were shown in Figure 6(C). All of the mice treated with RVG_29_-Nano-BA_P85_ survived for 14 days in an excellent physical condition. Treatment with RVG_29_-Nano-BA, Nano-BA_P85_, Nano-BA, and PEGylated Nano-BA_12K_ could also prolong the mean survival time similar to that in the Penicillin G group. Similar results were obtained for *S. pneumonia* 16167 infected PM mice (Figure 6(D–F)). All of the tested Nano-BAs significantly suppressed the growth of *S. pneumonia* 16167 compared with the negative control. Among the tested Nano-BAs, RVG_29_-Nano-BA_P85_ demonstrated the strongest *in vivo* antibacterial potency as characterized by the highest bacterial eradication and survival rate. Penicillin G demonstrated similar antibacterial efficiency as saline did, indicating a failure of the therapy.

### 3.8 Nephrotoxicity evaluation

To evaluate the safety of RVG_29_-Nano-BA_P85_, sections of kidney were stained with hematoxylin and eosin. As shown in Figure S4, glomerulosclerosis and tubulointerstitial damage was clearly observed in the kidney sections of the free BA-treated group. In contrast, there was no injury found in the kidney of the RVG_29_-Nano-BA_P85_-treated groups in both *S. pneumonia* ATCC 49619 and *S. pneumonia* 16167 infected PM mouse models, indicating low nephrotoxicity and good safety *in vivo*.

## 4. Discussion

Pneumococcal meningitis is a large public health concern, especially in children and immunocompromised patients. Although the epidemiology of Pneumococcal meningitis has shown significant decline in past decades, partly due to the introduction of vaccines, outbreaks are still reported worldwide. Our preliminary study indicated that PEGylated Nano-BA_12K_ showed excellent antibacterial potency against *S. pneumonia* with good biocompatibility. In this study, the possibility of using PEGylated Nano-BA_12K_ to treat Penicillin-sensitive and Penicillin-resistant pneumococcal meningitis was investigated. Formulating drugs to transverse the BBB is a necessary step in the design of anti-meningitis drug delivery systems. For brain-targeting delivery of PEGylated Nano-BA_12K_, a mixed micellar system formulated with a brain-targeting ligand of RVG_29_ and P-gp inhibitor of Pluronic^®^ P85 unimers (RVG_29_-Nano-BA_P85_) was constructed. A small proportion of RVG_29_-PEG-PLGA-PEG-RVG_29_ impart the capability of the mixed micelles crossing the BBB through receptor-medicated transcytosis (RMT) (Liu et al., 2009; Banks, 2012; Kim et al., 2013). Pluronic^®^ P85 unimers can inhibit the P-gp drug efflux system to further increase the permeability of BBB.

First, the composition of RVG_29_-Nano-BA_P85_ was determined. In order to brain-targeting deliver of PEGylated Nano-BA_12K_, the mixed micelles were conjugated with RVG_29_. The cellular uptake of PEGylated Nano-BA_12K_ was enhanced by ligand modification, which was respectively owing to the specific binding to nAchR highly expressed on BCECs and then initiated RMT process. However, high receptor-density (>5%) impeded cellular uptake, which might be due to the receptor saturation. In addition, considering that a larger amount of RVG_29_-PEG-PLGA-PEG-RVG_29_ would also influence the antibacterial activity, integrity and stability of RVG_29_-Nano-BA_P85_ in serum, 5% RVG_29_ modified copolymers was selected to construct RVG_29_-Nano-BA_P85_. Furthermore, it should be noted that the loading content of Pluronic^®^ P85 unimers also had a remarkable effect on the cellular uptake of RVG_29_-Nano-BA_P85_. The cellular uptake firstly increased with the increase of Pluronic^®^ P85 loading content until reaching 0.1 wt%. When the loading content was higher than 0.1 wt%, the cellular uptake first decreased and then leveled off. We speculated it was correlated with the different existence form of Pluronic^®^ P85 unimers in the mixed micelles. When the loading content of Pluronic^®^ P85 was less than 0.1 wt%, Pluronic^®^ P85 unimers randomly inserted into the mixed micelles. This could result in a much easier release of Pluronic^®^ P85 unimers to block the P-gp function. When the loading content above 0.1 wt%, a triple-component mixed micelles were formed by Pluronic^®^ P85, BA-PEG-PLGA-PEG-BA, and RVG_29_-PEG-PLGA-PEG-RVG_29_. The more ordered micellar structure and stronger interaction with other copolymers could generate the physical barrier and impede the release of Pluronic^®^ P85 unimers from the mixed micelles. Similar phenomenon was also observed by our previous research (Hong et al., 2013). Thus, the mixed micelles RVG_29_-Nano-BA_P85_ consisting of 5 wt% RVG_29_-PEG-PLGA-PEG-RVG_29_ (0.5 mg/mL), 95 wt% BA-PEG-PLGA-PEG-BA (9.5 mg/mL), and 0.1 wt% Pluronic**^®^** P85 (0.01 mg/mL) with excellent endocytosis ability and antibacterial activity were prepared for further studies.

The *in vitro* antibacterial activities assay revealed a comprehensive result that all of the Nano-BAs were effective against both Penicillin-sensitive and Penicillin-resistant *S. pneumonia* stains. In addition, Penicillin-resistant *S. pneumonia* was more susceptible to Nano-BAs loading with P85 unimers (RVG_29_-Nano-BA_P85_ and Nano-BA_P85_) than those without P85 unimers (RVG_29_-Nano-BA and Nano-BA). Comparatively, for Penicillin-sensitive strains, there was no obvious difference in MICs between Nano-BAs loading with and without P85 unimers. We speculated that P85 unimers might have the potency to reverse the bacterial resistance. Recently, there is overwhelming evidence indicating that Pluronic block copolymers were the most promising MDR reversal agent against cancer cells due to their reversal effect on several distinct drug resistance mechanisms (Venne et al., 1996; Evers et al., 2000; Yamagata et al., 2007). Basically, it can block drug efflux transporters, such as P-glycoprotein (P-gp), through exhausting the ATP pools and decreasing membrane fluidization. Nowadays, resistance to first-line antimicrobial agents complicates therapy of infections. A component of the resistance can be ascribed to the activity of membrane-based efflux transporters. Primary active transporters utilize ATP energy to move substrates. Pluronic^®^ P85 unimers might have a similar reversal effect on bacterial efflux transporters through depletion of the intracellular ATP levels and decrease of cell membrane fluidization. However, less is known on the reversal mechanism of Pluronics against bacterial resistance. Whether or not the presence of Pluronics may change the energy conservation and membrane fluidization of drug-resistant bacteria, and consequently trigger the intracellular reversal mechanism is a topic that remains to be studied in our future investigation.

For effective meningitis treatment, drugs must be accumulated in brain parenchyma. However, the effective transport of Nano-BAs to the brain parenchyma is still a problem. The BBB is composed of a layer of endothelial cells together by tight junctions, thus restricting the passage of substances (particularly water-soluble molecules or molecules larger than Mr =200 ∼ 400) from the bloodstream. In general, there are two possible pathways for facilitating molecules to cross the BBB: specific receptor-mediated endocytosis and adsorptive endocytosis routes (Chen & Liu, 2012). In this study, receptor-mediated transcytosis and P-gp inhibitor were combined simultaneously to develop a BBB crossing mixed micellar drug delivery system. *In vitro*, RVG_29_ and P85 unimers showed a synergetic effect on cellular uptake and transport across a BBB monolayer, being beneficial from receptor-mediated transcytosis, ATP exhaustion and membrane microviscosity reduction. In addition, the present findings obtained by the *in vivo* optical imaging study further provided incontrovertible evidence of the preferable targeting effect of dual-functional micelles (RVG_29_-Nano-BA_P85_) at all time points and relatively long residence at the brain tissue in PM mice. Previous studies suggested that *in vivo* biodistribution of nanoparticles are partly determined by their own surface properties and loading content. Nanocarriers functionalized with targeting ligands have been frequently reported for overcoming BBB, and then moving into the CSF and diffusing into the brain tissues. RVG_29_ modified Nano-BAs could significantly enhance the accumulation of PEGylated Nano-BA_12K_ in brain tissues through receptor-mediated endocytosis. Moreover, formulating with Pluronic P85 unimers could further facilitate the penetration of PEGylated Nano-BA_12K_ through the BBB by blocking the P-gp function. Moreover, RVG_29_-Nano-BA_P85_ resulted in limited fluorescence signal in kidney, indicating that a high target efficiency and long circulation time could significantly reduce the accumulation in kidney, leading to low toxicity. *In vivo* antibacterial activity data demonstrated that RVG_29_-Nano-BA_P85_ monotherapy effectively suppressed the growth of both Penicillin-sensitive and -resistant *S. pneumonia* in the brain with invisible side effect, consistent with the *in vitro* results. Many attempts have been made to combine receptor-mediated endocytosis and Pluronics reversal agents to MDR cells using micellar delivery systems due to their ability to self-assemble into micelles. For example, Wang and Zhang et al. developed folate-mediated Pluronic^®^ P105 micelles and Pluronic^®^ F127/P123 mixed micelles to overcome MDR, respectively. The authors indicated that combination of folate-mediated active targeting and Pluronics MDR reversal effect could enhanced the paclitaxel accumulation in both the resistant breast cancer MCF-7/ADR cells and resistant ovarian cancer SKOV-3/PTX cells, leading to enhanced cytotoxicity against MDR cells. Few researches focused on the combination of active-targeting and Pluronics P-gp inhibitor for brain-targeting delivery system. In this study, we formulated RVG29 and P85 to brain-targeting deliver of PEGylated Nano-BA_12K_. The results indicated that RVG_29_-Nano-BA_P85_ represents a suitable candidate of safe and efficacious nanomedicines in future clinical applications against both Penicillin-sensitive and -resistant PM.

## 5. Conclusions

In summary, we designed and fabricated mixed micelles (RVG_29_-Nano-BA_P85_) formulated with RVG_29_ and P85 unimers for efficient delivery of PEGylated Nano-BA_12K_ to brain tissues against both Penicillin-sensitive and -resistant pneumococcal meningitis. Based on the *in vitro* cellular uptake and antibacterial assay results, a formulation of 5 wt% RVG_29_-PEG-PLGA-PEG-RVG_29_ (0.5 mg/mL), 95 wt% BA-PEG-PLGA-PEG-BA (9.5 mg/mL), and 0.1 wt% Pluronic**^®^** P85 (0.01 mg/mL) was developed for the BBB targeting and crossing mixed micellar drug delivery system. As expected, RVG_29_-Nano-BA_P85_ demonstrated high BBB-crossing ability that compared with single formulated micelles. The excellent brain-targeting performance featured by a higher receptor binding affinity and a lower P-gp function, leading to a satisfactory accumulation in the brain and a high therapeutic efficacy of both Penicillin-sensitive and -resistant PM with negligible systemic toxicity. In brief, based on those finding, RVG_29_-Nano-BA_P85_ is a promising alternative for the treatment of brain infections with sensitive and resistant *S. pneumonia*. The design of nano-antiseptic drugs aiming at the active-targeting and P-gp inhibition provides a new potential way to tackle the brain delivery bottleneck.

## Supplementary Material

Supplementary Materials
