# Supplementary material for: Brain-targeted delivery of PEGylated nano-bacitracin A against Penicillin-sensitive and -resistant Pneumococcal meningitis: formulated with RVG29 and Pluronic® P85 unimers
Source: Drug Deliv. 2018 Nov 7;25(1):1886–97. doi: 10.1080/10717544.2018.1486473 (PMC6225518; doi:10.1080/10717544.2018.1486473)
Supplement: Supplementary Materials [file IDRD_A_1486473_SM6921.docx]

*Supplementary Materials for*

**Brain-Targeted Delivery of PEGylated Nano-bacitracin A against Penicillin-sensitive and -resistant Pneumococcal Meningitis: Formulated with RVG_29_ and Pluronic^®^ P85 unimers**

Wei Hong, Zehui Zhang, Lipeng Liu, Yining Zhao, Dexian Zhang, Mingchun Liu^*^

*Key laboratory of Zoonosis of Liaoning Province, College of Animal Science and Veterinary Medicine, Shenyang Agricultural University, Dongling Road 120, Shenyang, Liaoning Province, 110866, P.R. China*

^*^ *Corresponding author. Tel./Fax.: +86-24-88487156*

*E-mail address: Liumingchun@sina.com*

**1. METHODS**

**1.1 Synthesis and Characterization of RVG_29_-PEG-PLGA-PEG-RVG_29_**

RVG_29_-PEG(2K)-PLGA(12K)-PEG(2K)-RVG_29_ was synthesized by conjugating HO- PEG(2K)-PLGA(12K)-PEG(2K)-OH to the cysteine residue on RVG_29_. In brief, the triblock copolymers HO- PEG(2K)-PLGA(12K)-PEG(2K)-OH and N,N’-Carbonyldiimidazole (CDI) were dissolved in 15 mL of dry acetonitrile, respectively. The CDI solution was added dropwise to the solution of HO- PEG(2K)-PLGA(12K)-PEG(2K)-OH at room temperature over 2 h under nitrogen atmosphere. After addition, the mixture was kept stirring for an additional 24 h under nitrogen atmosphere. The solution was concentrated in a rotary evaporator, and pour into excess of ethyl ether. This process was repeated three times to remove unreacted CDI. The CDI-PEG(2K)-PLGA(12K)-PEG(2K)-CDI was dried for 3 days under vacuum and collected as white powder. The CDI-activated PEG(2K)-PLGA(12K)-PEG(2K) and RVG_29_ (molar ratio 1:2) were dissolved in anhydrous DMSO. Mixtures were gently stirred for 24 h at room temperature. Excessive peptides were removed by dialysis against distilled water. Pure products were freeze-dried and stored at -20 ºC until use.

^1^H-NMR spectra were recorded on a Bruker DRX-600 NMR instrument operated at 600 MHz and were used to determine the molecular structure and composition of the intermediates and final copolymer of RVG_29_-PEG(2K)-PLGA(12K)-PEG(2K)-RVG_29_. DMSO-*d*_6_ and CDCl_3_ were used as the solvent, including 0.03 vol.-% tetramethylsilane (TMS).

The molecular weight and molecular weight distribution of the intermediates and final copolymers were measured by a gel permeation chromatography (GPC) system equipped with a Waters 515 HPLC Pump, a waters StyragelTM HT3 column (300×7.8mm) and a Waters 2414 refractive index detector. Tetrahydrofuran (THF) was used as eluent with a flow rate of 1 mL/min at 35 ºC. The concentration of the samples was 10 mg/mL. The molecular weight of the copolymer was determined relative to polystyrene standards.

The critical micelle concentration (CMC) of the RVG_29_-PEG-PLGA_12K_-PEG-RVG_29_ was determined with a fluorescence technique using pyrene as a probe.[^1^](#_ENREF_1) Aliquots of pyrene solution (2.4×10^−5^ M, 50 μL) in acetone were poured into phosphate buffered saline (PBS) solution, and the acetone was eliminated by stirring at 40 ºC for 6 h. After the solvent was evaporated, 10 mL of the aqueous copolymer solutions with concentration ranging from 1×10^-4^ g/L to 1×10^-1^ g/L were added. The final pyrene concentration in the copolymer solution was 1.2×10^-7^ mol/L. All the solutions were stored in the dark place for 24 h to reach solubilization equilibrium prior to measurements. The fluorescence spectra of pyrene were recorded on a RF-5301 fluorescence spectrophotometer (Shimadzu Spectrofluorophotometer, Japan). The intensity ratio (I_385_/I_374_) of the pyrene emission spectrum was plotted as a function of polymer concentration at the excitation wavelength of 350 nm.

**1.2 *In vitro* cytotoxicity**

The *in vitro* cytotoxicity of RVG_29_-Nano-BA_P85_, Nano-BA_P85_, RVG_29_-Nano-BA and Nano-BA against BCECs and HK-2 cells were assessed by a standard thiazolyl blue tetrazolium bromide (MTT) assay, respectively. Briefly, BCECs and HK-2 cells were seeded in 96-well plates at the density of 5×10^3^ cells/well and incubated overnight, respectively. The growth medium was then replaced with fresh containing an indicated concentration of the tested formulations, respectively. Control wells were treated with equivalent volume of blank medium. After incubation for 48 h, the wells were rinsed with PBS, and then replaced with MTT solution (5 mg/mL). The plates were further incubated for 4 h at 37 ºC, allowing the viable cells to reduce the yellow MTT into purple formazan crystals. At last, the medium was removed completely and 150 μL of dimethyl sulphoxide (DMSO) was added to each well to dissolve purple formazan crystals. The absorbance was measured with at 570 nm using a multifunctional microplate reader (Tecan, Austria). The IC_50_ values were calculated using nonlinear regression analysis.

**2. RESULTS**

**2.1 Characterization of intermediates and final copolymers**

The ^1^H NMR spectra of the intermediates and the final copolymers were shown in Fig. S1A-D. All the chemical shifts were expressed in parts per million (δ) relative to the solvent signal. The ^1^H NMR spectrum (DMSO-d_6_) of RVG_29_ showed peaks at δ_a_ 10.45 ppm (-NH_2_), and δ_b_ 3.81 ppm (-COOH) (Fig. S1A), which was in good correlation with the previous study.[^2^](#_ENREF_2) The ^1^H NMR spectrum (DMSO-d_6_) of PEG-PLGA_12K_-PEG showed peaks at δ_a_ 5.19 ppm (-COCH(CH_3_)O-), δ_b_ 4.87 ppm (HO-CH_2_-), δ_c_ 3.51 ppm (-CH_2_-CH_2_-) and δ_d_ 1.48 ppm (-COCH(CH_3_)O-) (Fig. S1B). The ^1^H NMR spectrum (DMSO-d_6_) of CDI-PEG-PLGA_12K_-PEG-CDI showed peaks at δ_d_ 7.19 ppm (the proton “d” on the imidazole moiety), δ_e_ 8.48 ppm (the proton “e” on the imidazole moiety), δ_f_ 7.81 ppm (the proton “f” on the imidazole moiety) as well as the characteristic peaks of PEG-PLGA_12K_-PEG (Fig. S1C). The ^1^H NMR spectrum (DMSO-d_6_) of final copolymer RVG_29_-PEG-PLGA_8K_-PEG-RVG_29_ showed the characteristic peaks of PEG-PLGA_12K_-PEG at δ_a_ 5.21 ppm (-COCH(CH_3_)O-), δ_d_ 1.48 ppm (-COCH(CH_3_)O-), δ_c_ 3.37 ppm (-CH_2_-CH_2_-) as well as the characteristic peaks of RVG_29_ at δ_b_ 3.81 ppm (Fig. S1D). The disappearance of carboxyl group peak form RVG_29_ and hydroxyl group peak form PEG-PLGA-PEG suggesting the final triblock copolymer of RVG_29_-PEG-PLGA_8K_-PEG-RVG_29_ was obtained.


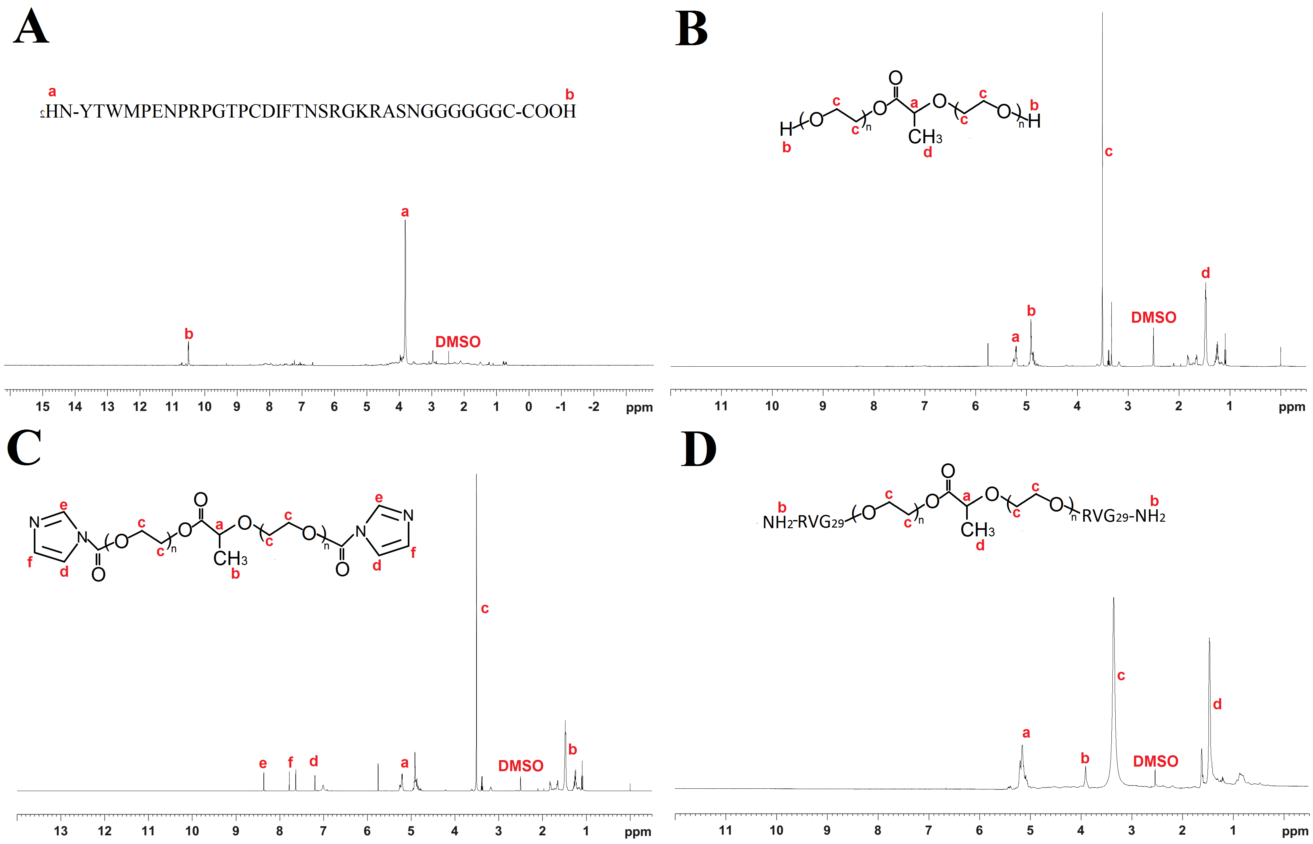


**Fig. S1** ^1^H NMR spectrum of RVG_29_ (A), PEG-PLGA-PEG (B), CDI-PEG-PLGA-PEG-CDI (C) and RVG_29_-PEG-PLGA-PEG-EVG_29_ (D).

The GPC chromatograms of PEG-PLGA_12K_-PEG and RVG_29_-PEG-PLGA_12K_-PEG-RVG_29_ were shown in Fig. S2. All the copolymers showed unimodal distribution with a polydispersity of <1.2, it was reasonable to assume that most of the copolymers have single structure. The shorter elution time of copolymers indicated an increase in the molecular weight, proving the successful conjugation of bacitracin A to copolymer.


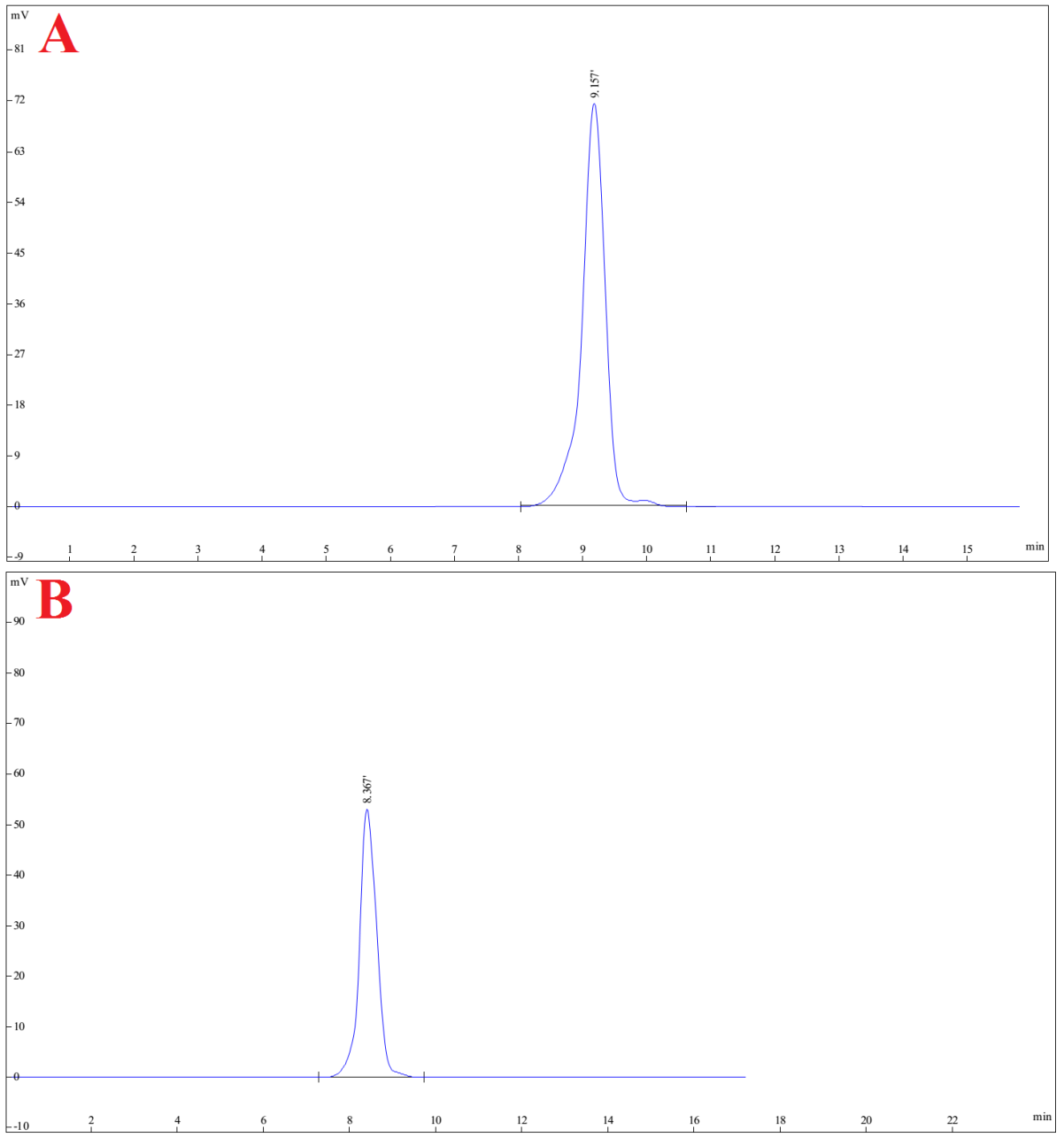


**Fig. S2** The typical GPC spectrum of PEG-PLGA_12K_-PEG (A) and RVG_29_-PEG-PLGA_12K_-PEG- RVG_29_ (B)

The CMC of RVG_29_-PEG-PLGA_12K_-PEG-RVG_29_ at biological pH (pH 7.4) was investigated using the pyrene-based fluorescent probe method. Pyrene is highly hydrophobic and preferentially migrates into the hydrophobic micellar core when it locates in the micellar solution. When pyrene is located in a a hydrophobic micellar core, it shows stronger florescence. The CMC values of the RVG_29_-PEG-PLGA_12K_-PEG-RVG_29_ copolymers and RVG_29_-Nano-BA_P85_ were 4.7 µg/mL and 12.2 µg/mL at pH 7.4, respectively.

The mean particle sizes of various formulations ranged from 116 to 127 nm, while PDIs were less than 0.1(Fig. S3).


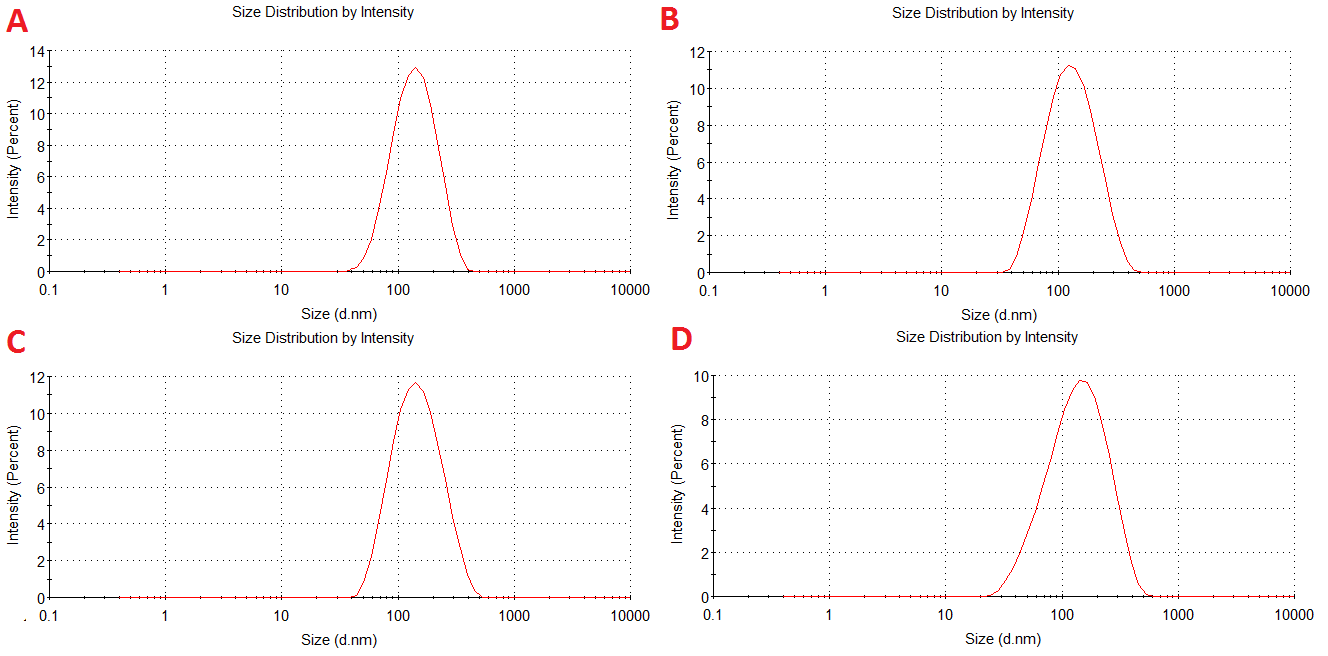


**Fig. S3** Particle size distribution of RVG_29_-Nano-BA_P85_ (A), RVG_29_-Nano-BA (B), Nano-BA_P85_ (C) and Nano-BA (D) determined by DLS.

**2.2 *In vitro* cytotoxicity**

BA is not intended for systemic administration because it is nephrotoxic. To elucidate whether PLGA induced self-assembly could reduce the cytotoxicity of BA, *in vitro* cytotoxicity of the RVG_29_-Nano-BA_P85_, Nano-BA_P85_, RVG_29_-Nano-BA and Nano-BA against HK-2 cells were measured by MTT assay (Table S1). Compared to BA solution, all of the Nano-BAs exhibited higher IC_50_ value against HK-2 cells after 24 h incubation, proving lower cytotoxicity. However, after incubation for 48 h, the IC_50_ values of Nano-BAs significantly decreased, implying the cytotoxicity increased with the prolongation of incubation time. Furthermore, there was no obvious difference on IC_50_ values of different Nano-BAs, indicating that small amount of RVG_29_-PEG-PLGA-PEG-RVG_29_ and Pluronic^®^ P85 did not influence the cytotoxicity of Nano-BAs. All of the Nano-BAs exhibited low cytotoxicity against BCECs, representing as high IC_50_, which was much higher than their MICs.


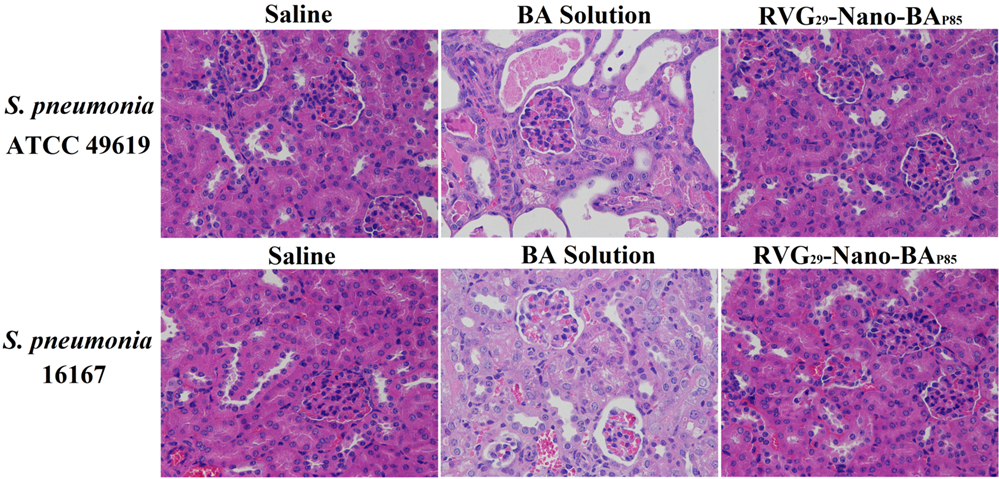


**Fig. S4** Histochemistry inspection of kidney stained with hematoxylin and eosin of PM mice infected *S. pneumonia* ATCC 49619 or *S. pneumonia* 16167 after treated with saline, BA solution, RVG_29_-Nano-BA_P85_, respectively. Original magnification=200×.

**Tables**

**Table S1** IC_50_ values of BA solution and Nano-BAs against HK-2 cells and BCECs (n=3)

| Formulations | HK-2 IC_50_ (μM) | | BCECs IC_50_ (μM) | |
| --- | --- | --- | --- | --- |
|  | 24 h | 48 h | 24 h | 48 h |
| BA Solution | 4.36±0.47 | 4.18±0.29 | 43.15±0.98 | 39.54±0.87 |
| RVG_29_-Nano-BA_P85_ | 31.02±0.74 | 6.39±0.64 | 44.22±0.39 | 38.22±0.81 |
| Nano-BA_P85_ | 30.88±0.61 | 6.28±0.33 | 43.87±0.41 | 39.45±0.29 |
| RVG_29_-Nano-BA | 30.76±0.59 | 6.21±0.47 | 44.01±0.56 | 40.01±0.71 |
| Nano-BA | 30.54±0.86 | 6.17±0.91 | 43.92±0.76 | 39.01±0.45 |

**Table S2** The physicochemical characterization of mixed micelles (n=3)

| Formulations | Particle size (nm) | ξ potential (mv) | PDI |
| --- | --- | --- | --- |
| RVG_29_-Nano-BA_P85_ | 126.6±1.19 | -4.01±0.05 | 0.069±0.007 |
| RVG_29_-Nano-BA | 117.8±1.02 | -4.04±0.06 | 0.078±0.005 |
| Nano-BA_P85_ | 124.2±1.23 | -4.29±0.11 | 0.082±0.009 |
| Nano-BA | 116.0±1.31 | -4.31±0.13 | 0.093±0.008 |

**Table S3** Antibacterial activities of the tested formulations (MIC)

| Strain | MIC^a^ (µM) | | | | | | |
| --- | --- | --- | --- | --- | --- | --- | --- |
|  | RVG_29_-Nano-  BA_P85_ | RVG_29_-Nano-BA | Nano-BA_P85_ | Nano-BA | PEGylated Nano-BA_12K_ | BA | Penicillin G |
| 49619 | 0.5 | 0.5 | 0.5 | 0.5 | 0.5 | ˃128 | 0.125 |
| 16033 | 1 | 2 | 1 | 2 | 2 | ˃128 | 64 |
| 16055 | 0.5 | 0.5 | 0.5 | 0.5 | 0.5 | ˃128 | 2 |
| 16067 | 0.5 | 0.5 | 0.5 | 0.5 | 0.5 | ˃128 | 0.125 |
| 16089 | 0.5 | 0.5 | 0.5 | 0.5 | 0.5 | ˃128 | 1 |
| 16092 | 1 | 4 | 1 | 4 | 4 | ˃128 | 32 |
| 16113 | 1 | 1 | 1 | 1 | 1 | ˃128 | 0.125 |
| 16121 | 0.5 | 1 | 0.5 | 2 | 2 | ˃128 | 16 |
| 16124 | 0.5 | 0.5 | 0.5 | 0.5 | 0.5 | ˃128 | 0.125 |
| 16129 | 1 | 4 | 1 | 2 | 4 | ˃128 | 32 |
| 16145 | 1 | 1 | 1 | 1 | 1 | ˃128 | 1 |
| 16167 | 1 | 4 | 1 | 8 | 8 | ˃128 | ˃128 |
| 16192 | 0.5 | 1 | 0.5 | 1 | 2 | ˃128 | 2 |

^a^ Minimal inhibitory concentrations (MICs) were determined as the lowest concentration of the tested copolymers that inhibited bacterial growth.

**Reference**

(1) Maiti, S., Chatterji, P. R., Nisha, C. K., Manorama, S. V., Aswal, V. K. and Goyal, P. S. Aggregation And Polymerization Of Peg-Based Macromonomers With Methacryloyl Group As The Only Hydrophobic Segment. *J Colloid Interface Sci*. **2001,** *240*, 630-635.

(2) Ming, L. J. and Epperson, J. D. Metal Binding And Structure-Activity Relationship Of The Metalloantibiotic Peptide Bacitracin. *J Inorg Biochem*. **2002,** *91*, 46-58.
